# Development of the Digestive System—Experimental Challenges and Approaches of Infant Lipid Digestion

**DOI:** 10.1007/s13228-012-0025-x

**Published:** 2012-11-07

**Authors:** Evan Abrahamse, Mans Minekus, George A. van Aken, Bert van de Heijning, Jan Knol, Nana Bartke, Raish Oozeer, Eline M. van der Beek, Thomas Ludwig

**Affiliations:** 1 Centre for Specialised Nutrition, Danone Research, P.O. Box 7005, 6700 CA Wageningen, The Netherlands; 2 TNO, Zeist, The Netherlands; 3 NIZO food research, Ede, The Netherlands; 4 Centre for Specialised Nutrition, Danone Research, Singapore, Singapore

**Keywords:** Breast milk, Gut development, Infant nutrition, Lipase, Bile salts, Gastric lipase, PUFA, Fat, Lipids, Digestion, In vitro simulation, Obesity, Cholesterol, Phospholipids, Metabolic syndrome, Metabolic programming

## Abstract

At least during the first 6 months after birth, the nutrition of infants should ideally consist of human milk which provides 40–60 % of energy from lipids. Beyond energy, human milk also delivers lipids with a specific functionality, such as essential fatty acids (FA), phospholipids, and cholesterol. Healthy development, especially of the nervous and digestive systems, depends fundamentally on these. Epidemiological data suggest that human milk provides unique health benefits during early infancy that extend to long-lasting benefits. Preclinical findings show that qualitative changes in dietary lipids, i.e., lipid structure and FA composition, during early life may contribute to the reported long-term effects. Little is known in this respect about the development of digestive function and the digestion and absorption of lipids by the newborn. This review gives a detailed overview of the distinct functionalities that dietary lipids from human milk and infant formula provide and the profound differences in the physiology and biochemistry of lipid digestion between infants and adults. Fundamental mechanisms of infant lipid digestion can, however, almost exclusively be elucidated in vitro. Experimental approaches and their challenges are reviewed in depth.

## The Developing Digestive System

Birth constitutes a dramatic switch in the supply of nutrients from the placenta to the gut and, with the first feeding, the exposure of the gastrointestinal system to something different from amniotic fluid. This transition generates the necessity to digest macronutrients prior to their absorption and makes the gut, with its large, folded surface, our biggest interface to the outside world. In essence, very little is known about the complex development of digestive functions in human newborns. This is not surprising, since the clinical investigation of digestive processes frequently requires invasive procedures, such as the usage of nasogastric and nasoduodenal tubes or the drawing of blood samples. Their invasiveness limits the applicability of such procedures in healthy term infants. Preterm infants are usually fed via a gastric tube that also allows the collection of samples. As a result, lipid digestion has, to the best of our knowledge, only been functionally investigated in preterm infants, but not term infants [12, 128].

Lipids comprise a broad group of small molecules that, besides fats (here defined as triacylglycerols (TAGs)), include other hydrophobic or amphiphilic compounds, such as phospholipids (PLs), monoacylglycerols and diacylglycerols (MAGs and DAGs), and sterols. The functionality of these has been reviewed elsewhere in detail [83, 84]. However, from stool samples, it can be concluded that lipids are not completely absorbed by infants. The amount of fat that is excreted with the stool can approximate 10 % in term infants and 10–30 % in preterm infants [9, 14, 37, 38, 42, 91, 138, 157]. The amount of unabsorbed fat appears to depend essentially on gestational and postnatal age and the type of fat [126]. This is likely to be linked to the maturation of the digestive system, be it the digestive function (i.e., enzyme and bile salt (BS) levels), absorptive capacity, or a combination of both.

## Infant Lipid Digestion and Microbiota Composition

Unabsorbed lipids are transported to the colon where they might influence or be utilized by the colonic microbiota. The latter has been extensively credited for its relevance in health and disease. It provides nutrients and energy through the fermentation of dietary and endogenous components [43]. During the first year of life, the dietary influence on the development of microbiota composition and functionality has been extensively studied. As such, human milk oligosaccharides have been identified to be critical for shaping the gut’s microbiota composition [27, 82]. In infants, the specific effect of unabsorbed long-chain fatty acids (LCFA) on the colonic microbiota is likely to be masked by the continuous supply of prebiotic oligosaccharides. It has actually been demonstrated that the effect of fish oil on infant’s microbiota is only detectable after ceasing human milk feeding [7]. Although the impact of a high-fat diet on human or rodent microbiota has been widely documented, the influence of undigested LCFA on microbiota function in infants remains to be elucidated [7, 80, 118, 140].

## Lipids in Human Milk and Infant Formula

**Fig. 1:**
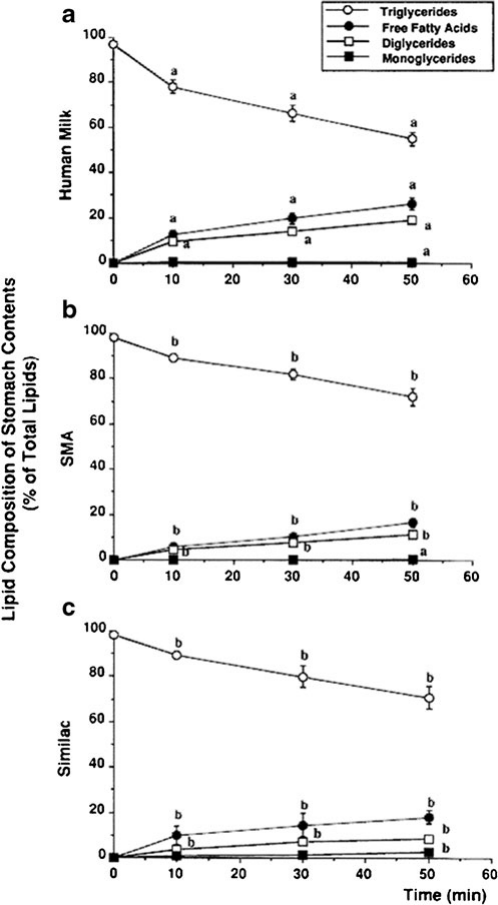
Gastric lipolysis was investigated in breast fed (**a**), and two groups of formula fed preterm infants ((**b**) SMA SP and (**c**) Similac SC). Data are given as mean±SE. *Different superscript letters* indicate significant differences. From [12]

Interestingly, the aforementioned fecal fat excretion has been found to be higher in infant formula (IF)-fed infants than in breast-fed infants [14, 42]. In addition, it was concluded in two studies that gastric TAG hydrolysis from human milk is 1.7 to 2.5 times higher than that of IF, while gastric pH, emptying, and enzyme output were not different in these infants [12, 128] (Fig. 1). It is likely that most of these findings are related to the differences in the quality and composition of lipids in human milk compared to IFs.

**Fig. 2:**
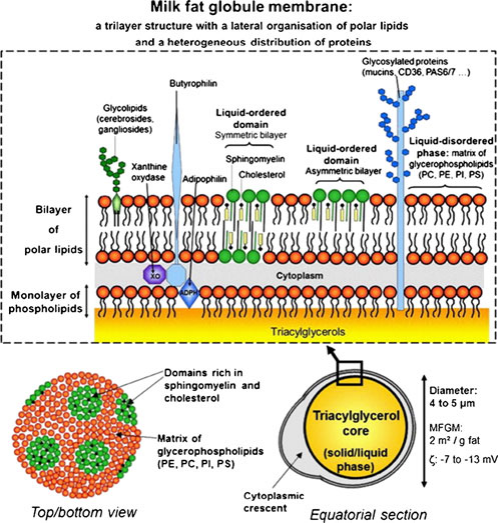
Structure of the human milk fat globule membrane (MFGM). A trilayer of polar lipids forms the backbone of the MFGM. (*PC* phosphatidylcholine; *PE* phosphatidylethanolamine; *PS* phosphatidylserine; *PI* phosphatidylinositol). From [93]

Lipids are intricately packed in human milk. Human milk fat is secreted as milk fat globules (MFGs) from the alveolar epithelial cells of the lactating mammary gland. The human MFGs have a typical diameter of 3–9 μm. MFGs consist of a hydrophobic core comprising mainly of TAGs, which is enveloped by a triple layer of amphipathic compounds, such as PLs, proteins, including enzymes, and cholesterol, which together assemble the milk fat globule membrane (MFGM) [84, 94, 110, 114, 129] (Fig. 2). A comparison of the lipids in mature human milk and those in current IFs shows differences in fatty acid (FA) composition and in their physical structure. The fat droplets in IF are usually much smaller in size, about 0.4 μm, and lack the membrane envelope [110, 137].

The content and composition of human milk fat varies and has been found to depend among other factors on the mother’s diet and the stage of lactation. Human milk total lipid content increases during lactation from 2 g/dL in colostrum to 4.9 g/dL in mature milk and adapts to the needs of the growing child [102]. Human milk has an average total lipid content of about 3.8–3.9 g/100 ml. About 98 % of human milk’s total lipid content is TAGs, which serve mainly as an energy source for the fast-growing infant. The remaining fat is a complex mix of polar lipids, including PLs, glycolipids and sphingolipids, fat-soluble vitamins, cholesterol and cholesterol esters, and small amounts of lipolysis products, such as free fatty acids (FFAs), MAGs, and DAGs. These lipids deliver important functional materials, such as the building blocks for cellular membranes in various tissues, antioxidants, and signaling molecules. In human milk, polar lipids account for 0.8–2.2 %, and cholesterol for about 0.5 % of the lipids. The various polar lipid species include sphingomyelin (37.5 %), phosphatidylcholine (28.4 %), phosphatidylethanolamine (27.7 %), phosphatidylserine (8.8 %), and phosphatidylinositol (6.1 %) [84].

The TAG composition of human milk fat resembles that of mature adipose tissues. Hence, it contains a ratio of saturated over unsaturated fat that is appropriate for cell membrane function and has a melting point around body temperature [32]. The main FAs found in human milk are palmitic acid (PA, C16:0, 26.5 %) and oleic acid (OA, C18:1 n-9, 35.5 %). The essential FA linoleic acid (LA, 18:2 n-6, 7.2 %) is the most abundant polyunsaturated fatty acid (PUFA), followed by the other essential PUFA α-linolenic acid (ALA, 18:3 n-3, 0.8 %) [102]. In recent years, the long-chain polyunsaturated fatty acids (LCPUFAs) arachidonic acid (AA, 20:4 n-6, 0.3–1.0 %) and docosahexaenoic acid (DHA, 22:6 n-3, 0.1–0.9 %) have been credited as important functional components in human milk [78]. These LCPUFAs are not only necessary for normal brain development, with its peak from the last trimester of pregnancy and the first year(s) of life, but also for immune function [33, 83]. Average n-6 to n-3 ratios of 5 to 10 have been observed in human milk. These increased up to a ratio of 18 if corn, sunflower, or safflower oils were enriched in the mother’s diet. The AA:DHA ratio is usually between 1:1 and 2:1. Eicosapentaenoic acid (EPA) is only found in minimal amounts in human milk, except for populations with high sea fish intakes [78]. Initially, IFs were not supplemented with LCPUFAs as it was assumed that infants could synthesize these FAs from their precursors and young infants appear to be able to convert ALA to DHA and LA to AA [57]. In neonates, the enzymatic activity to form these LCPUFAs may nonetheless be too limited to meet, for example, the high requirements of these building blocks for the developing neural tissue. Therefore, supplementation of IFs with LCPUFAs is now widely recommended.

A relatively large proportion (15–20 %) of LCPUFAs in human milk is provided through the PL fraction, although most of the LCPUFAs are esterified to TAGs because of the considerably higher total amount of the latter [65]. Hence, differences in the physicochemical properties of LCPUFAs linked to either TAGs or PLs suggest different absorption mechanisms and deviating biological functions. Consequently, the dietary source of LCPUFAs could be of biological significance for the developing infant. Direct evidence for this is given in nonhuman primates. It has been shown that dietary AA-PL is at least as efficacious as dietary AA-TAG to supply the developing organs with AA [155]. No direct information relates to the effects of DHA-TAG versus DHA-PL in this context [2, 5, 6, 38, 58, 106, 155].

Various FAs have a preference for their linkage to the glycerol core molecule in a specific position. This has been found to depend on their carbon atom chain length and their degree of saturation. In human milk TAGs, the most abundant saturated FA (PA) exhibits a high preference for the sn-2 position of 60–70 %. The sn-1 and sn-3 positions of human milk TAGs are commonly occupied by unsaturated FAs, such as OA and LA, as well as medium-chain FAs (mainly 12:0). Contrarily, today, most IFs contain much higher amounts of PA in the sn-1 or sn-3 position of the TAGs [139]. This difference in the localization of a specific FA in the TAGs might be crucial for the metabolization of dietary fat by infants. For instance, it has been shown that, due to a decreased calcium soap formation, fat and calcium absorption can be improved in IFs containing PA in the sn-2 position, compared to IFs with PA in the sn-1 or sn-3 position [37, 90].

## Lipid Architecture and Nutritional Programming

Human milk has a highly complex lipid architecture. Human epidemiological studies that compare breast-fed and formula-fed infants suggest a moderate protective effect of human milk against rapid growth during the first year after birth and the risk of cardiovascular and metabolic disease later in life [131]. It was hypothesized that the specific surface composition and size of the lipid droplets in human milk may contribute to the reported long-term benefits of breastfeeding.

To test this hypothesis, a concept IF was developed with lipid droplets that exhibit physical properties similar to those in human milk, i.e., larger in size and coated with PL (Nuturis®) and tested in a mouse model of nutritional programming [120, 143]. Studies showed comparable body weight (BW) and gain in absolute and relative fat mass between Nuturis® and control groups directly after early diet intervention. The postnatal Nuturis®-containing diet reduced, however, the increase in fat mass gain by a subsequent Western-style diet challenge by 30 % [121]. These results suggest that qualitative changes in dietary fat in early life, such as fat structure and FA composition, may have a significant contribution to the early development of body composition [120]. This knowledge on the functionality of fat quality in the early diet may contribute to an improved understanding of childhood overweight and obesity risks and may help to develop a preventative strategy for future generations. The lasting effects of fat globule architecture in nutritional compositions during infancy on metabolic functions later in life illustrate the complexity of the subject matter. These effects might ultimately be linked to changes in digestion due to changes in physical structure.

## Physiology and Enzymology of Lipid Digestion in Infancy

Human milk is relatively rich in lipids. Surprisingly, this does not match with the relatively low levels of pancreatic TAG lipase (PTL) and BSs that one may expect to be required for their digestion [161]. The digestive physiology of newborns deviates from that of adults beyond the smaller size of the different digestive organs [1, 81]. Based on distinct requirements, the quality and quantity of enzymes and bile, pH values, gut permeability, and meal and eating patterns differ considerably between infants and adults. As such, infant lipid digestion relies on lipases other than PTL, such as gastric lipase (GL), bile salt-dependent lipase (BSDL, also called carboxyl esterase lipase), and pancreatic lipase-related protein 2 (PLRP2) [8, 64, 91]. Moreover, human milk also contains bile salt-stimulated lipase (BSSL), which is an enzyme with close similarity to the pancreatic BSDL [91].

Compared to adults, gastric lipid digestion has a more prominent function in young infants. Lipolysis by GL starts in the stomach after ingestion [41]. In newborns, GL activity (units per volume) is found to be slightly lower or comparable to that of adults fed a high-fat diet, which may also depend on the used activity assay [11, 12, 128]. GL is secreted by the chief cells in the fundus of the stomach and has a preference for FAs on the sn-3 position of the TAG. This stereo-preference results in an apparent specificity for short-chain FAs, as the short-chain and medium-chain FAs are located on the sn-3 position in milk TAGs [18, 55, 127, 128]. GL has a high activity in a broad pH range. Its pH optimum is at a pH between 5.4 and 5.8, and the enzyme is resistant to low pH values. The acidification in the infant’s stomach is slower compared to that of adults, which brings the gastric pH closer to the optimum of the lipase [63, 128, 132, 147]. The enzyme retains part of its activity in the small intestine which broadens its contribution to lipid digestion [4, 39, 89, 128]. GL activity can account for 10–30 % of dietary TAG hydrolysis in adults and up to 60 % of dietary TAG hydrolysis in infants [39, 41, 62, 128, 141]. However, FFAs that are protonated at the low pH in the stomach can also form layers adsorbed onto the fat droplet, which limits gastric lipolysis [56, 122]. The FFAs generated in the stomach by GL can furthermore suppress microbial growth in absence of efficient acidification by hydrochloric acid [77].

While the pH of the stomach declines, the mixture of digestive juices and food, now called chyme, is emptied into the duodenum. The gastrointestinal tract reacts to the acidic and nutrient-rich chyme with the aim to neutralize its pH, to control the entry of nutrients, and to activate the secretion of bile and pancreatic fluid [18]. The latter contains a broad range of enzymes and bicarbonate, which neutralizes the acidic gastric effluent. Pancreatic digestive enzymes found in infants are among others (1) *amylolytic* (α-amylase), (2) *proteolytic* (trypsin, chymotrypsin, and elastase), and (3) *lipolytic* (PTL, BSDL, PLRP2, and phospholipase A2 (PLA2)) [26, 60, 61, 87, 95, 107]. As the name indicates, BSDL is activated by bile, while it does not need colipase for activation. PLRP2 has activity against TAGs, PLs, and galactolipids [46]. Its activity is influenced by colipase [158]. PTL does not hydrolyze the ester bond at the sn-2 position of TAG. BSDL and PLRP2 contrarily have no preference for the sn-1, sn-2, or sn-3 position of the FA [91, 150]. Lipolysis by these enzymes may thus result in only FFAs and glycerol. The digestive lipases operate synergistically in the digestion of emulsified fat. Hence, the liberation of FAs by GL in the stomach contributes to the onset of lipolysis by pancreatic colipase-dependent lipases and BSDL [18, 19, 56].

PLA2 hydrolyzes PLs that cover TAG droplets into the more polar and surface-active monoacyl PL. This releases FFAs, which turn the triglyceride droplet into a better substrate for PTL [30]. This synergy is of direct importance for the digestion of MFGs [31]. Also, BSDL and PLRP2 have been shown to have a synergistic effect on TAG hydrolysis [8].

## The Lipid Paradox and Functionality of Bile Salts

The capacity to digest fat is not only incomplete and suboptimal at birth due to low pancreatic enzyme levels as mentioned before, but also due to low intraluminal BS concentrations. Yet, lipolysis and nutrient lipid absorption in the term, breast-fed infant are remarkably efficient by adult standards, considering the amount of lipid an infant consumes. In essence, several mechanisms have evolved to “circumvent” this relative immaturity of the pancreatic, intestinal, and hepatobiliary systems.

BSs are highly relevant for the dispersion, enzymatic activity, and solubilization of the hydrolysis products into an absorbable form, with an inadequate supply to the gut resulting in suboptimal lipid absorption. Intraluminal BS levels above the critical micellar concentration (CMC; ∼4 mM) are required for a proper absorption of dietary lipids and/or lipophilic compounds. Hence, a critical BS mass is required for optimal fat absorption [152].

At birth, the human fetus switches from a glucose-dominated to a lipid-dominated energy supply. The proper digestion and absorption of lipid is hence important, not only to meet energy requirements but also for optimal growth and neuronal development [91]. Fat intake in newborns (2.5–3.5 g/kg BW/day) is three to five times higher compared to adults. It seems, therefore, a paradox that the main enzyme involved in TAG digestion in adults, i.e., PTL, is virtually absent at birth. Also, luminal BS levels reach only 1–5 mM during established fat absorption, whereas in adults, levels of up to 30 mM can be reached after a fatty meal. The CMC of BSs, needed for solubilization of the products of lipid digestion and its subsequent absorption, can thus hardly be reached during infancy. Hence, both digestion and absorption are expected to be different (i.e., lower) from adults in the newborn. However, despite this paradox, fat absorption in newborns fed with unpasteurized human milk is more efficient than one would expect [8, 91, 151].

Bile is very important for the absorption of fat and lipophilic substances. Although up to about 50 % of fat can be absorbed in the absence of bile, this fat is then absorbed as FFAs, predominantly through direct portal absorption and avoiding the lymphatic route that is normal for long-chain TAG [29, 54]. Moreover, the FAs tend to accumulate in the small intestinal mucosa [54, 125]. The absorption of other lipid-soluble substances, such as cholesterol, vitamin D, vitamin K, and carotene, are almost completely dependent on the presence of bile [116, 130, 136]. Bile is secreted from the liver and temporarily stored and concentrated in the gall bladder. It is a mixture of mainly BSs, PLs, cholesterol, and bicarbonate. Bile’s constituents are highly surface-active, which is essential for its function as an emulsifier for any non-emulsified fat that enters the small intestine and to ensure a large accessible surface area for the lipases [70, 72, 73]. Moreover, the BSs and PLs form micelles. By solubilization, these micelles are able to remove lipolytic reaction products and other emulsifiers from the droplet surfaces, which would otherwise form a surface barrier for the lipases. These micelles are also needed for PTL. The active site of PTL is covered by a molecular lid that can only be fully opened by forming a complex with its colipase and appropriate micellar species, such as bile micelles, and in solution possibly also requires the presence of a lipid/water interface [16, 67, 124]. Finally, the BS micelles can transport the released MAGs and FAs through the mucous lining of the small intestine, making them available for absorption [99, 141]. This is especially important for the LCFA and MAGs because they are insoluble in water at body temperature [99].

Compared to adults, infants have a different composition of their BS pool. Secondary BSs are virtually absent in newborns which lack an established gut flora and hence substantial 7-dehydroxylation. In contrast to adults, BSs in infants are mainly taurine-conjugated [71]. BSs are synthesized from cholesterol by the hepatocytes. Despite an increased rate of synthesis in infants, the BS pool size is smaller due to both incomplete intestinal recovery and hepatic extraction. This results in considerably higher plasma BS levels in infants which are until an age of 6–8 months typically three to ten times higher than in adults [71, 152].

About 50 % of the FAs in human milk are saturated; these require BSs for their solubilization and subsequent absorption [101]. Thus, infants with bile acid malabsorption may run into digestive problems regarding their lipid dietary intake.

## Bile Salt Transport and Enterohepatic Recycling

Ileal active BS transport is absent at birth and develops during the first postnatal weeks and was shown in the rat to develop during the third postnatal week [69]. The active BS-transporting capacity increases with age, which was suggested to relate to a change in the microvillus membrane lipid composition (more unsaturated FA) at unchanged PL levels and a decrease in membrane fluidity due to an increased cholesterol content. To what extent dietary effects contribute to this association remains to be elucidated [69].

As active BS transport is compromised and confined to the distal ileum in early life, enterohepatic BS cycling is less efficient. Despite of this, no excessive BS loss in stool is observed, although relative to their BW, fecal BS loss in infants may be higher or similar to that of adults [71]. Apart from BSs, the stool of breast-fed infants consists mainly of bacteria and unabsorbed FA, as no substantial amounts of insoluble fibers are ingested.

The postnatal development of the enterohepatic circulation entails an increase in the BS pool, which correlates with increased BS levels in the gut and with an improved fat absorption. The increased BS pool size leads to an efficient absorption as lipolysis, solubilization, and absorption of dietary lipid are best facilitated. The observation that fat absorption from fresh human milk is markedly more efficient than from formula milk might be directly related to the finding that breast-fed infants show a larger BS pool and higher intraluminal BS concentrations compared to formula-fed infants, both at 11 and 35 days of age [152]. The recycling frequency of BS was shown to be a major determinant of BS pool size: a reduced recycling frequency, for example, due to a slower bowel transit time, has been shown to increase the BS pool [152]. Hence, a rapid small bowel transit would quickly return BS to the liver and in this way reduces the stimulus for BS synthesis. The latter mechanism might be operational in the breast-fed infants. It is not clear if this mechanism of stimulated BS synthesis by a reduced BS recycling frequency explains or contributes to the observed larger BS pool and the more efficient fat absorption in breast-fed infants compared to formula-fed infants [144]. Hence, the maturation of fat absorption in human neonates is possibly linked to the developmental changes which are observed in BS composition and pool size.

## Changes in Enzyme and Bile Acid Functionality During Maturation

With increasing age towards weaning and the introduction of solid foods, BS secretion increases due to liver function maturation and PLRP2, BSDL, and BSSL become less important to TAG digestion. This coincides with PTL becoming the dominant TAG lipase. The increasing intraluminal BS levels attenuate PLRP2 and BSDL activity as both enzymes are BS-sensitive. At the same time, the higher intraluminal BS levels enable an efficient FA absorption [8].

However, until weaning, with milk as the main feed and PLRP2 acting together with BSSL, the synergistic effect of the combination is likely to compensate BS levels to maintain neonatal capacity to digest fat. Fat-digestive capacity is thus safeguarded, which would otherwise become a limiting factor in energy utilization [8].

## Bile Salt-Stimulated Lipase and Lipid Architecture Facilitate Lipid Digestion

As stated before, digestion and absorption of fat from human milk has been found to be more efficient compared to that of IF. Digestion is a result of the interdependence of the nutritional composition and the corresponding hydrolytic enzymes. In this context, human milk may facilitate the digestibility of its lipids via distinct mechanisms.

To support the absorption of TAG, cholesterol, and fat-soluble vitamins in infancy, human milk contains BSSL, which is very similar to BSDL. BSSL is inactive in the milk and in the stomach, but survives the stomach’s acidic and proteolytic environment. It is activated by bile in the small intestine. It has been shown in rats that BSSL binds to the intestinal mucosa and efficiently hydrolyses cholesterol esters, which improves the overall absorption of cholesterol [49].

In addition, the MFGM of human milk has a high PL content which may contribute to BSSL activation by bile. As BSSL is active on all positions of the TAG molecule, BSSL might also be functional in further hydrolyzing the DAG formed as the endpoint of GL digestion. However, the complex interplay of the different lipases, their substrates, and the infant’s digestive physiology remains to be clarified [148].

Lipolysis by the water-soluble lipases occurs usually in the presence of an emulsifier at the oil/water interface. The rate and extent of lipolysis depends, next to the presence of endogenous factors like PLA2, BSs, and colipase, also on the droplet characteristics. These include the area of the TAG surface available to the lipase, primarily determined by the droplet size, and on the emulsifier type [13, 17, 23, 24, 28, 109, 111]. However, the relation between these droplet characteristics and their digestion is anything but simple. Lipolytic activity is clearly increased when a higher accessible surface area is available; however, the emulsifier appears to be of at least equal importance [21, 109, 111]. In human milk, this “emulsifier” is the MFGM, consisting of PL and membrane proteins, whereas in IF, this is usually only protein. The results from in vivo gastric aspirates studies suggest that GL activity could be affected by the droplet’s size and interface composition of the MFG, as BSSL is unlikely to be active under gastric conditions [12, 128]. Although emulsifiers could potentially influence the lipolysis rate by inhibiting or promoting the access of the enzyme to the TAGs, this effect seems to be relatively small [123].

Dependent on the emulsifier type, declining pH and enzymatic activity can destabilize the emulsion droplets, which results in flocculation of the droplets [103–105, 142]. This could lead to a fat layer on top of the gastric contents that delays the entrance and detection of fat in the small intestine [53, 74, 103, 104, 154]. It has been shown that, although PL-stabilized emulsions strongly aggregate under gastric conditions, native cow milk MFGs remain dispersed under gastric conditions of lipolysis [123]. Apparently, the protein structure of the MFGM can keep the fat droplets separated, without inhibiting gastric lipolysis. Furthermore, for intact cow’s milk, it was found that the MFGs flocculate together with the caseins to form a soft gel in the stomach, which sediments due to its high protein content [142]. Whether this is also applicable for human milk, with its higher fat and lower protein content, remains to be elucidated.

## Lipolytic Enzymes for the In Vitro Study of Lipid Digestion in Infancy

In vitro approaches, which have been built on the available clinical data on luminal biochemistry, offer an opportunity to generate insights in infant digestion. Based on the complexity of both the substrate and the involved lipolytic enzymes, the in vitro investigation of lipid digestion during infancy has proven particularly challenging.

In order to realistically simulate the lipid digestion of the newborn, GL, PTL, BSDL, and PLRP2 should be present in physiological amounts. However, this approach is challenged by the fact that human GL and PLRP2 are not commercially available. Furthermore, commercial pancreatin may not contain PTL, BSDL, and PLRP2 in concentrations that are adapted to human infant levels [149]. It has been found that porcine pancreatic extracts do contain BSDL [22]. However, levels of BSDL and PLRP2 in infants are not exactly known [8].

Active human GL has been successfully produced in insect and yeast cells [4, 35]. However, the yield with these methods appeared to be too low for commercial exploitation. Recombinant dog GL was produced in transgenic plants at high levels [59]. However, the cultivation of genetically modified plants has been a controversial subject in the public opinion. As a result, 40,000 m^2^ of GL-expressing corn that were grown to substitute cystic fibrosis patients were destroyed in 2003 [98].

Due to the lack of availability of human GL, several alternative enzymes have been evaluated. Fungal lipases, such as F-AP15 from *Rhizopus orizae* (Amano Enzyme Inc.) and lipase A “Amano” 12 from *Aspergillus niger* (Amano Enzyme Inc.), are, for example, active at a low pH. However, these enzymes do not have the same positional and FA chain length specificity as the human GL. In addition, their activity might be high also at neutral pH, thus having a larger contribution to lipolysis in the small intestine as compared to human GL. Commercially available pregastric or lingual lipase from lamb or calf, which are used to hydrolyze fat and produce a specific flavor in some cheeses, may offer alternative lipase sources [20]. These enzymes belong to the acid lipase gene family like human GL and are structurally and functionally similar. They are active at lower pH values and also have a preference for the sn-3 position. However, the FA chain length specificity and the pH activity profile are different, and they are not as acid-stable as human GL [117]. Recently, approaches to obtain sufficient quantities of GL were reported. For instance, rabbit GL has been proposed as a replacement for human GL [36]. In addition, the human GL has been successfully expressed in *Nicotiana benthamiana*, a close relative of the common tobacco plant [145].

It can be concluded that the simulation of lipid digestion under infant conditions with realistic enzyme activities is hampered by the availability of GL and PLRP2 and the lacking knowledge on the exact activities of PLRP2 and BSDL in the infant. The question is whether or not it is necessary to precisely reproduce these enzyme activities to study infant lipid digestion. In essence, there is no simple answer as this will always depend on the research question at hand. For example, it has been shown that the specific activities of GL and PTL may result in a higher availability of PA that is predominantly present at the sn-2 position of human milk fat [90]. Even though GL contributes very little to sn-2 lipolysis and PTL activity in infants is low, intestinal lipolysis can be taken over by BSDL and PLRP2, which are capable of complete TAG hydrolysis. However, although GL may not have the most important contribution from a quantitative point of view, GL’s stereospecificity has functional implications and it has also been proposed to be relevant for the action of pancreatic lipases [56, 90, 119].

BSDL and PLRP2 have specific activities that could be crucial for the investigation of lipids other than TAGs. BSDL is a carboxyl esterase with activity against a variety of substrates, such as TAGs, cholesterol esters, galactolipids, ceramides, and PLs [52, 92]. PLRP2 not only hydrolyses TAGs but also PLs and galactolipids [46, 48]. Given this overlap in substrate specificity, BSDL could be used as an approximation to the in vivo conditions as the main pancreatic lipase if PLRP2 is not available. However, BSDL does not appear to be able to hydrolyze the MFG by itself as BSDL and PLRP2 work synergistically [8].

## In Vitro Models Mimic Digestive Physiology

Two cardinal types of in vitro models—static and dynamic—can be distinguished, with a broad range of intermediates that can be further divided according to their approach. In static models, the addition of digestive juices occurs in one bolus. The mixing of chyme and digestive juices usually occurs in a single compartment by stirring. More realistic simulations of digestion processes are obtained in dynamic models, in which the secretion of digestive juices is gradual. In some dynamic models, peristaltic movement can also be simulated. In advanced models, various physiological units are combined together in consecutive order, e.g., the stomach, the small intestine, and the colon. Advanced dynamic models may also remove products of digestion from the intestinal lumen to simulate absorption and prevent product inhibition of the enzymes.

Lipid digestion is most often measured by means of chemical quantification of FFAs by titration or by chromatography. The measurement of TAG digestion products by, e.g., mass spectroscopy or in situ densitometric analysis yields more detailed insights [134]. Radiolabeled substrates are also used to improve the identification of products formed [79].

Optical microscopy, ζ-potential, cryo-transmission electron microscopy, and particle size analysis can give mechanistic insight into the physical nature of lipolysis [108]. The recent introduction of multiplex coherent anti-Stokes Raman scattering microspectroscopy has brought a promising new means to continuously image the digestion process [45].

Lipid digestion models have been frequently used to investigate, for example, the interaction of oral drugs with foods, digestion of lipid-based drug delivery systems, and controlled digestion of emulsion-based systems in the research of satiety [15, 47, 50, 86, 108].

### Single-Phase Static Models

The pH-stat method is the most widely used method to investigate lipid digestion [88, 108]. The method is based on measurements of the amount of FFA released from lipids (usually TAG) after lipase addition at a fixed pH. The method usually simulates the conditions within the small intestine with physiological amounts of PTL, colipase, BSs, and minerals. The reaction takes place in a single thermostatically controlled vessel. The release of FFAs as a result of lipolysis will subsequently liberate protons that would acidify the vessel. The pH is kept constant by continuous titration with a base, and the amount of base needed is proportional to the amount of FFA released. The method has similarities to traditional lipase activity assays [39]. Most FFAs have a high p*K*_a_ value, which can make them partly ionized and titratable under assay conditions. Therefore, back titration at the end of the experiment at high pH is often needed for the estimation of total FFA formed [51, 156].

This relatively simple method allows the investigation of the impact of different factors on the rate of lipolysis by PTL, without the complicating effects of pH variation, gradual dilution, and gradual increase in the enzyme concentration. Factors that have been studied among others are the type of TAG, amount of calcium, BS dependency, droplet size, and emulsifier type [3, 10, 28, 44, 68, 75, 76, 134, 135].

The titration method provides no details on individual lipid digestion products as the measured response is independent from the type of FFA. It has been shown that a combination of the pH-stat method with gas chromatography analysis not only enabled the monitoring of MAG and DAG concentrations, but also demonstrated that the amount of FFA released during lipolysis could be underestimated in emulsions stabilized by whey protein isolate [66].

FFA released during lipolysis can accumulate on the emulsion interface and therewith inhibit PTL by a process called product inhibition [34, 133]. In vivo, solubilization of FFA in BS-PL micelles together with MAG is believed to be the most effective way of removing the FFA from the emulsion interface [160]. Continuous addition of calcium has been shown to prevent product inhibition in vitro, probably through the precipitation of released FFA [50, 159]. This addition extends the dynamic range of the method. The efficacy of FFA removal will, however, depend on the FA type. Moreover, a too efficient binding of FFA to calcium ions might change the properties of the bile micelles, which during in vivo digestion become filled with the released lipolytic hydrolysis products. A different approach to prevent FFA accumulation on the emulsion interface is the addition of FFA-free bovine serum albumin to the reaction medium [28].

Since the pH-stat method usually focuses on small intestinal lipolysis, being the main locus of lipid digestion in adults, the processes that take place in the stomach phase are usually ignored. It has been shown, however, that the complete digestion of human milk fat in vitro requires also GL [19]. In addition, the influence of gastric lipolysis on small intestinal lipolysis can be significant [19, 28, 56]. This applies especially to infants where the relative contribution of the intestinal phase to overall lipid digestion is lower due to the low PTL. An in vitro study of milk lipid digestion in infancy should, therefore, encompass a gastric phase before the intestinal phase.

### Sequential-Phase Static Models

A sequential approach in the pH-stat setting has been developed, in which distinct gastric and intestinal phases were incorporated [40]. Incubation with a gastric juice at pH 5.5 for half an hour was followed by incubation with a pancreatic juice/bile solution at pH 6.25 for an hour in this setting. The conditions used were based on the analysis of gastric and duodenal aspirates at 50 % of gastric emptying of a liquid preparation for enteral nutrition. The method is applied, for example, to study lipolysis of test meals containing various fats, to predict the efficacy of a lipase inhibitor, to study the effects of lipolysis on the solubility of hydrophobic drugs formulated with lipids, and recently, to compare the lipolytic potential of crude animal digestive lipases with that of human gastric and pancreatic juices and that of purified human digestive lipases [36, 41]. In this method, the analysis of FFA, MAG, DAG, and TAG was performed using thin-layer chromatography and flame ionization detection. The measurement of released FFA is, therefore, not dependent on titration. As a next step, this method has been combined with Caco-2 monolayers to investigate the intestinal absorption and lipid metabolism of the lipolysis products in the intestinal epithelium [8, 148]. It was found that the emulsion composition influenced the activation of lipid metabolism and TAG secretion by the epithelial cells.

Static model systems are usually designed dedicated to a specific research question and improve mechanistic interpretability by avoiding addition of parameters, such as gradual pH variation and dilution. However, since the number of simulated parameters is limited, their predictability towards the complex in vivo situation may remain limited. The sequential-phase model, however, has been shown to have predictive power towards the in vivo situation in the case of the action of a lipase inhibitor [41]. Static methods exclude per definition the dynamic addition of fresh digestive juices and the removal of digestion products, simulated digestive juices, and liquid volume. The proteases which are present in, for example, crude pancreatin can also degrade the lipase and other digestive enzymes.

### Dynamic Models

In vivo, the conditions change considerably along the gut in space and time. Mixing and propagation occurs by contractions of the gastric and intestinal walls, while the pyloric sphincter selectively allows food components to pass based on feedback mechanisms and particle size. This causes the gastric content to be gradually emptied from the stomach. During gastric residence, the meal is mixed and diluted with saliva and gastric secretions. The pH is not constant but increases due to the buffer capacity of the meal during intake, after which it decreases due to the secretion of gastric acid. Enzyme activities in time are the result of both their output and the gastric pH in time. While the gastric content enters the duodenum, it is mixed with bile and pancreatic secretions. The gastric pH is neutralized by bicarbonate to accommodate duodenal enzyme activities and bile action, while changing the ionic state and solubility of molecules. This is crucial for the formation of mixed micelles, comprising of BSs, FAs, MAGs, PLs, and cholesterol, which provide a transport vehicle preceding the absorption of lipophilic compounds. Besides providing the body with nutrients, the removal of digestive products by transport through the gut wall prevents the inhibition of enzyme activity by their digestive products. Water is also reabsorbed, which concentrates the chyme.

**Fig. 3:**
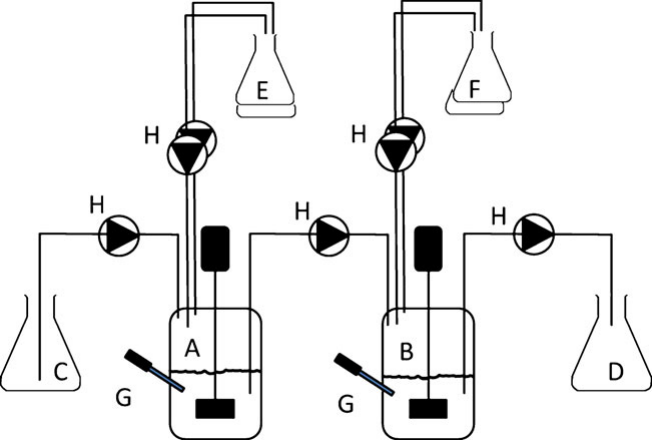
Example of a dynamic system with two stirred vessels. *A* stomach vessel, *B* small intestine vessel, *C* influent vessel, *D* effluent vessel, *E* gastric secretions with acid and enzymes, *F* duodenal secretions with alkali, bile, and pancreatic enzymes, *G* pH electrodes, *H* pumps

Dynamic models aim to simulate these processes more realistically than static models, which make them more complex and labor-intensive. However, this is well compensated by their higher predictive power and broader applicability. The basic dynamic models are based on a series of stirred vessels connected by pumps (Fig. 3). These models have been designed to study antacids [146], the survival of probiotics, and the interaction of ingested compounds with the intestinal microbial ecosystem [100, 115]. Gastric dynamic models were designed to study the gastric behavior of food and pharmaceuticals with realistic physical and mechanical aspects. The dynamic gastric model (DGM) [153] consists of a funnel-shaped vessel that mimics the upper part of the stomach. Mild mixing is obtained by slow contractions of a flexible membrane that acts as the gastric wall. The antral motility is mimicked with a plunger system that mixes and grinds the chyme before emptying from the gastric compartment. In the DGM, F-AP15 (Amano Enzyme Inc.) is used as GL substitute.

Another model that simulates the physical properties of the stomach is the human gastric simulator (HGS) [85]. This model creates peristaltic movements by moving rollers along a flexible artificial stomach wall. In the HGS, lipase A “Amano” 12 (Amano Enzyme Inc.) is used as GL substitute.

These gastric models are useful to study the gastric digestion and aspects, such as particle breakdown, rheology, phase separation, and release of nutrients and pharmaceuticals.

**Fig. 4:**
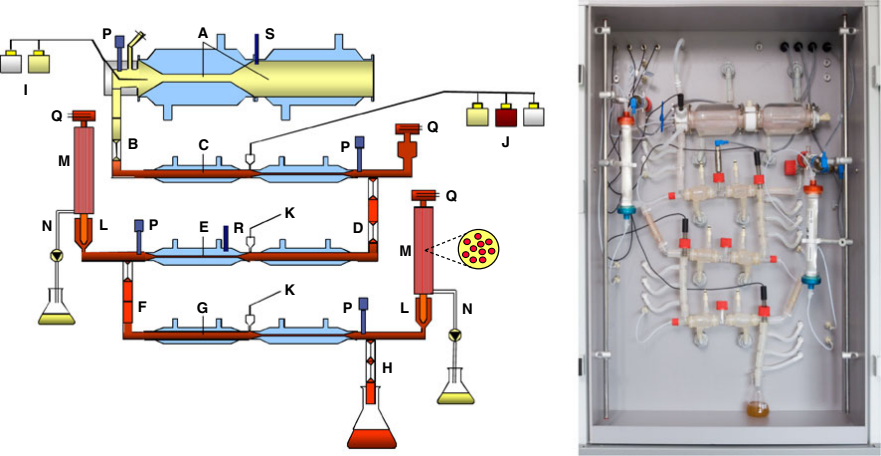
**a** Schematic representation of the TIM-1 lipid system. *A* gastric compartment, *B* pyloric sphincter, *C* duodenum, *D* peristaltic valve, *E* jejunum compartment, *F* peristaltic valve, *G* ileum compartment, *H* ileocecal valve, *I* gastric secretion, *J* duodenal secretion, *K* bicarbonate secretion, *L* prefilter, *M* filtration membrane, *N* filtration pump, *P* pH electrodes, *Q* level sensor, *R* temperature sensor, *S* pressure sensor. **b** the TIM-1 cabinet

Both the gastric and the small intestinal digestive processes are simulated in the TNO gastrointestinal model (TIM) (Fig. 4), which simulates the successive dynamic conditions in the gastrointestinal tract [113]. The system is controlled following specific digestive protocols that include parameters in time for meal transit, pH profiles in the different compartments, temperature, secretion of digestive fluids, and removal rate of water and digestion products. F-AP15 (Amano Enzyme Inc.) is used as a GL substitute, while pancreatin is used as a source of pancreatic enzymes. The secreted bile is obtained from slaughterhouse pigs.

It offers the possibility to simulate the GI conditions in infants and adults and study age-dependent digestion [25]. Although this model lacks a realistic gut wall that selectively transports compounds by active or facilitated transport systems, devices are included in the system to study the removal of digested and solubilized compounds from the GI lumen. A dialysis system removes water-solubilized molecules below a specific molecular weight cutoff. A filtration system removes lipid-soluble products and allows mixed micelles and small vesicles to pass. Lipid droplets are retained in the lumen of the intestinal compartments because they are larger than the filtration membrane’s pores. By selectively removing the products of lipid digestion and fat-soluble compounds, the digestibility of various fats and oils and the availability for absorption, e.g., bioaccessibility, of FAs, nutrients, e.g., fat-soluble vitamins, and pharmaceuticals can be studied [112]. Subsequently, by combining samples from the TIM system with intestinal cell lines or segments, intestinal absorption of the compound of interest can be studied.

## Conclusions

Human milk has been shown to have a direct impact on the development of the infant’s digestive system, which equally impacts the digestion processes of its primary substrate [96, 97]. However, the mutual interdependencies between both remain to be elucidated. To date, little is known about the digestion and absorption of milk TAG and lipids, such as PLs and cholesterol, which provide functionality beyond energy. As such, lipids are a key component of the healthy development of, e.g., body composition and the nervous and gastrointestinal systems. Clinical outcomes linked to the latter, which are modulated by fat quality and quantity, can only be tested in clinical trials. The basic mechanisms of infant lipid digestion, however, can almost exclusively be investigated by in vitro approaches. The peculiarities of the infant digestive system—which is significantly different from a miniature version of the adult digestive system—generate methodological challenges. For example, specific enzymes, such as human GL that are highly relevant for lipid digestion during infancy, are not yet readily available. Nonetheless, in vitro systems—ranging from static single phase to complex and dynamic, multicompartmental systems—can be adequate approximations of infant digestive conditions for specific applications. This will allow the investigation of relevant parameters of human milk lipid digestion during infancy, which may help to support the further improvement of IFs.

## Abbreviations

AA: Arachidonic acid
ALA: α-Linolenic acid
BS: Bile salt
BSDL: Bile salt-dependent lipase
BSSL: Bile salt-stimulated lipase
DAG: Diacylglycerol
DHA: Docosahexaenoic acid
EPA: Eicosapentaenoic acid
FA: Fatty acid
FFA: Free fatty acid
GL: Gastric lipase
IF: Infant formula
LA: Linoleic acid
LCFA: Long-chain fatty acids
LCPUFA: Long-chain polyunsaturated fatty acid
MAG: Monoacylglycerol
MFG: Milk fat globule
MFGM: Milk fat globule membrane
PA: Palmitic acid
PL: Phospholipid
PLA2: Phospholipase A2
PLRP2: Pancreatic lipase-related protein 2
PTL: Pancreatic TAG lipase
PUFA: Polyunsaturated fatty acid
TAG: Triacylglycerol
